# The IGF1 Signaling Pathway: From Basic Concepts to Therapeutic Opportunities

**DOI:** 10.3390/ijms241914882

**Published:** 2023-10-04

**Authors:** Haim Werner

**Affiliations:** Department of Human Molecular Genetics and Biochemistry, School of Medicine, Tel Aviv University, Tel Aviv 69978, Israel; hwerner@tauex.tau.ac.il

**Keywords:** insulin-like growth factor-1 (IGF1), IGF1 receptor, MAPK, nuclear translocation, p53

## Abstract

Insulin-like growth factor 1 (IGF1) is a peptide growth factor with important functions in multiple aspects of growth, development and metabolism. The biological actions of IGF1 are mediated by the IGF1 receptor (IGF1R), a cell-surface protein that is evolutionarily related to the insulin receptor (InsR). The effects of IGF1 are moderated by a group of binding proteins (IGFBPs) that bind and transport the ligand in the circulation and extracellular fluids. In mechanistic terms, IGF1R function is linked to the MAPK and PI3K signaling pathways. Furthermore, IGF1R has been shown to migrate to cell nucleus, where it functions as a transcriptional activator. The co-localization of IGF1R and MAPK in the nucleus is of major interest as it suggests novel mechanistic paradigms for the IGF1R-MAPK network. Given its potent anti-apoptotic and pro-survival roles, and in view of its almost universal pattern of expression in most types of cancer, IGF1R has emerged as a promising molecular target in oncology. The present review article provides a concise overview of key scientific developments in the research area of IGF and highlights a number of more recent findings, including its nuclear migration and its interaction with oncogenes and tumor suppressors.

## 1. Introduction

Insulin-like growth factor-1 (IGF1) was discovered in the late 1950s, when the mechanisms of action of growth hormone (GH, or somatotropin) were investigated in animal models [1]. These early studies provided evidence that sulfate incorporation into cartilage was not a direct action of GH, but, rather an indirect process that involves the biosynthesis of a serum-borne mediator termed ‘sulfation factor’, which is directly responsible for the anabolic activities of GH. The catalogue of activities of the sulfation factor extended in the following years to include stimulation of proline incorporation into collagen, uridine into RNA, thymidine into DNA, etc. Moreover, the huge span of activities associated with the sulfation factor was substantiated by studies showing that its growth-promoting actions were not restricted to cartilage but, in fact, involved additional tissues and organs, including muscle, adipose tissue, brain, etc [2].

The term ‘*somatomedin*’ was subsequently coined to emphasize the fact that this serum factor mediates the effects of somatotropin at somatic target organs [3,4]. Further biochemical analyses led to the identification of two tightly linked peptides with growth-promoting functions in the serum fraction [5,6]. These peptides were shown to display insulin-like activities in the diaphragm and to stimulate glucose incorporation in fat. However, the insulin-like activities of these molecules were not suppressed by antibodies against insulin [6,7,8]. Hence, the factors that were initially referred to as *sulfation factor*, *somatomedin*, and ‘*NSILA*’ (non-suppressible insulin-like activity) were eventually termed insulin-like growth factor-1 and insulin-like growth factor-2 (IGF1 and IGF2) [9,10]. A recent article by Miller et al. [11] summarized the history of the IGF system.

**Figure 1:**
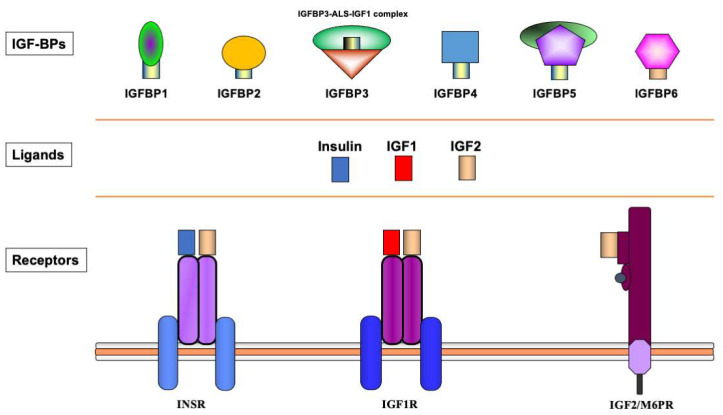
**Schematic representation of the insulin-IGF network**. The insulin-IGF family includes three ligands, three receptors and at least six IGFBPs. Experimental analyses provided evidence of cross-reactivity between ligands and receptors. Hence, IGF2 binds with high affinity to the InsR. In addition, both IGF1 and IGF2 activate hybrid receptors, formed by an IGF1R hemireceptor in association with an InsR hemireceptor (not shown). The IGF2/mannose 6-phosphate receptor is responsible for IGF2 recycling and is not involved in signaling. Most IGF1 in the circulation is bound to IGFBP3, which forms a multi-complex with an acid-labile subunit (ALS) and protects IGF1 from proteolytic degradation.

The biological actions of IGF1 and IGF2 are transduced by a group of transmembrane receptors that includes the insulin (InsR), IGF1 (IGF1R), and IGF2/mannose-6-phosphate (M6P) receptors [12,13,14,15]. While IGF1R is the primary mediator of IGF1/2 action, the InsR and IGF2/M6 receptor are also capable of mediating some of these activities, though with reduced affinities [16,17,18]. The IGF1R is linked to a series of intracellular pathways that includes the RAS-MAPK and PI3K-AKT signaling cascades [19]. The biological actions of the IGFs are moderated by a group of at least six IGF-binding proteins (IGFBP) that are produced by most tissues and are present in variable amounts in the circulation and extracellular compartments [20]. The IGFBPs are capable of inhibiting or enhancing IGF effects in a tissue-specific manner. Furthermore, various IGFBPs, most notably IGFBP3, also display ligand-independent effects [21,22]. A schematic representation of the major components of the IGF family is shown in Figure 1.

The *Somatomedin Hypothesis* was modified a number of times over the past decades, according to emerging scientific developments. It is widely accepted today that most, but not all, of the bioactivities of GH are mediated by liver-produced IGF1. While hepatic IGF1 biosynthesis is tightly dependent on GH stimulation, animal and human studies have identified a number of activities of IGF1 that are regarded as GH-independent.

The IGF network is recognized as a potential molecular target in cancer therapy and attempts are being made to translate preclinical results into medical protocols [23,24,25,26,27]. The aim of the present review article is to provide an updated overview of the IGF network, with a special emphasis on intracellular signaling events. In addition, we will discuss recent evidence regarding the nuclear localization of IGF1R. Finally, we will focus on the potential role of the IGF axis as an interventional target in oncology and other areas in which this hormonal axis is involved.

## 2. Insulin-like Growth Factor-1 (IGF1)

The *IGF1* gene, located on chromosome band 12q22–q24, encompasses at least 90-kb of chromosomal DNA and contains six exons [28,29,30]. Alternative transcription initiation and splicing and various polyadenylation sequences result in a number of IGF1 mRNAs [31]. These mRNAs slightly differ in their coding sequences but mainly diverge in their 5′ and 3′ untranslated portions [32,33].

The IGF1 peptide (~7.65-kDa) consists of 70 amino acids, while IGF2 (~7.47-kDa) contains 67 amino acids [10,33]. IGF1 and IGF2 display a 62% homology in their amino acid sequences and there is a 40% similarity between both IGF1/2 and proinsulin [34,35]. Unlike insulin, wherein the connecting C-peptide is cleaved out during prohormone processing, the mature IGFs retain the C-domain that links the A and B domains. These structural divergences may explain the immunological distinction between IGFs and insulin that led to the historical discovery of non-suppressible insulin-like activity of IGF1. Furthermore, IGFs contain an extension to the A domain, termed the D domain, which is not present in insulin. Finally, both IGF precursors contain C-terminal E peptides. These peptides are cleaved during the processing of the prohormone.

Low IGF1 and high IG2 levels are detected during the prenatal period in rodents. Postnatal stages are associated with an increase in circulating IGF1 concentrations and the disappearance of IGF2 [31,36]. These early findings might have led to a mistaken interpretation of the roles of IGF2 and IGF1 as fetal and pubertal growth factors, respectively [37]. In humans, however, IGF2 and IGF1 are produced from prenatal to postnatal periods. In fact, endocrine IGF2 levels in adults are higher than IGF1. Of importance, liver-specific *igf1* gene deletion in mice, while leading to a dramatic reduction in circulating IGF1 concentrations, had no major impact on body weight and length and femoral length. Hence, locally-produced (autocrine/paracrine) IGF1 seems to play a crucial role in organ and body growth and development [37,38].

In terms of the mechanisms that are responsible for regulation of the local production of IGF1, there is a marked variability between the different organs. In general, the biochemical machinery involved in IGF1 biosynthesis and action at the local level has been less well characterized than in the liver. Likewise, the paradigm that IGF1 production is tightly dependent on GH stimulation does not seem to apply to every tissue. Different tissue-specific hormones and growth factors have been shown to modulate IGF1 action. Thus, steroid hormones (e.g., androgens, estrogens) play a key role in IGF1 regulation in sex organs, while various neuropeptides control IGF1 activities in the brain.

The effect of polymorphisms in the *IGF1* gene on endocrine IGF1 levels and cancer risk is variable [39]. *IGF1* single-nucleotide polymorphisms (SNPs) individually account for up to 5% change in IGF1 concentrations, but no correlations have been observed between these polymorphisms and breast cancer risk. Hence, the impact of genetic variation in *IGF1* on IGF1 levels does not appear to substantially modify breast cancer risk.

## 3. Insulin-like Growth Factor-1 Receptor (IGF1R)

IGF1 and IGF2 bind to and activate a shared, ubiquitously expressed, transmembrane receptor, the IGF1 receptor (IGF1R). IGF1R signals mitogenic, pro-survival and anti-apoptotic activities [12,18,40]. The IGF2/M6P receptor does not seem to participate in IGF signaling, and its main role is to target IGF2 for proteolytic degradation at the lysosome [15]. The *IGF1R* gene is located on the long arm of chromosome 15 (15q25–q26), and spans more than 100 kb of genomic DNA [41]. The gene encodes a 1367-amino acid pre-pro-receptor that is processed to yield mature α and β chains [32]. The mature receptor has an heterotetrameric structure that includes two extracellular α-subunits, involved in ligand binding, and two transmembrane β-subunits, containing a tyrosine kinase domain in their cytoplasmic portion [42]. As described below in more detail, the IGF1R is linked to various cytoplasmic second messenger molecules. The RAS-MAPK and PI3K signaling networks are the most important players in this context.

IGF1R action is fundamental for survival, as demonstrated by the lethality of mice in which the *IGF1R* gene was inactivated [43]. The IGF1R is abundantly expressed at every ontogenetic period, beginning from the oocyte stage [44,45]. At late fetal stages and during adulthood there is a marked decline in IGF1R mRNA concentrations [46]. This decrease is inversely correlated with the high proportion of terminally differentiated cells at these stages. The crucial role of IGF1R in organ growth and development is exemplified by the fact that *IGF1R* gene disruption results in animals that weigh 45% of their control littermates at the moment of birth. These animals display many developmental defects: hypoplasia, delayed bone development, defective skin formation, and atypical central nervous system morphology, etc. These animals die from respiratory collapse immediately after birth.

Further evidence for the role of IGF1R in development and growth is provided by the fact that chromosomal alterations involving the 15q26 locus (e.g., ring chromosome 15) are correlated with hemizygosity of the *IGF1R* locus and growth deficit [47]. Conversely, a patient with three copies of the *IGF1R* gene that resulted from duplication of the long arm of chromosome 15 had an height and weight above the 97^th^ percentile. The patient exhibited an accelerated development [48]. These clinical studies highlight the link between IGF1R abundance and cell proliferation. Finally, analyses of multiple tumors showed high expression of IGF1R mRNA and protein. These tumors included breast, prostate, ovary, colon, hematopoietic, kidney, etc. These analyses led to the concept that *IGF1R* gene upregulation constitutes a common paradigm in cancer [49,50,51].

## 4. IGF Binding Proteins (IGFBPs)

Most of the IGF1 peptide in the blood is present in a ternary complex that includes, in addition to IGF1, a liver-produced glycoprotein (the acid-labile subunit, ALS) and an high-affinity-binding molecule, the IGFBP3 [20,52]. The proportion of free (or active) IGF1 is very low. At least six IGFBPs (IGFBP1–6) and a number of IGFBP-related molecules have been identified [53]. The predominant binding protein in blood is IGFBP3. Given its large molecular size, IGFBP3 cannot traverse the capillary membrane. The ternary complex formed by IGF1, IGFBP3 and ALS moderates IGF1 action by protecting the growth factor from proteolysis. As a result, IGF1’s half life is prolonged. In addition, as mentioned above, certain IGFBPs elicit their activities in an IGF-independent manner [22]. These findings are important in that they suggest that the spectrum of biological activities of IGFBPs goes beyond the characterized interactions with the IGF axis.

While the IGFBPs, in general, inhibit IGF actions, some IGFBPs display IGF-potentiating effects too [54,55]. IGFBP3 is regarded as an inhibitor of proliferation, eliciting a pro-apoptotic effect. A number of putative mechanisms have been postulated to explain IGFBP3 inhibitory activity. These mechanisms include sequestration of IGF1 from the receptor and binding competition with IGF1R [21]. In the specific case of prostate cancer, serum IGFBP2 was more than eight-fold higher in patients with metastatic disease compared to controls [56]. In contrast, a marked reduction in serum IGFBP3 was detected in patients with metastatic cancer. A significant correlation between serum IGFBP2 and prostate specific antigen (PSA) levels was observed, with a negative correlation between serum PSA and IGFBP3. These results suggest that IGFBPs participate in the growth regulation of prostate malignancy, and that variations in their blood levels may constitute biomarkers for prostate cancer.

A discussion on the complex activities of IGFBPs would be essentially impossible under the constraints of this review. A recent article by L. Bach provided an updated overview of IGFBPs [21]. IGFBPs control important biological processes such as proliferation, senescence, autophagy, migration, and angiogenesis. Furthermore, a number of mechanisms that are responsible for IGFBPs’ actions have been described, including modulation of other growth factors’ actions, transcriptional control, interaction with the sphingolipid pathway, binding to non-IGF molecules in the extracellular matrix, nuclear transport, etc. More studies are needed to evaluate the therapeutic potential of IGFBPs.

## 5. Signal Transduction

Ligand binding induces conformational changes that lead to autophosphorylation of the IGF1R β-subunit tyrosine kinase domain (comprising amino acids 973–1229) and subsequent ubiquitination of the receptor [57,58]. The IGF1R kinase domain contains an activation loop that includes three tyrosine residues (Tyr1,131, Tyr1,135 and Tyr1,136) that serve as autophosphorylation sites. Tyr1,135 and Tyr1,131 phosphorylation destabilizes the auto-inhibitory conformation of the activation loop, whereas Tyr1,136 phosphorylation stabilizes the catalytically optimized conformation. This step allows for substrate and ATP access [12,59]. Furthermore, the C-terminal domain includes a number of additional tyrosine and serine residues, such as Tyrs 1250, 1251 and 1316 and Sers 1280–1283. Phosphorylation of these sites is important in the context of IGF1R signaling. Mutation of all or some [60] of these residues affects the enzymatic activity as well as the biological properties of IGF1R [61,62]. The phosphorylated tyrosine residues serve as docking elements for other signaling molecules such as insulin receptor substrate (IRS)1-4 and Shc adaptor proteins. This event leads to activation of the PI3K/MAPK and the 14-3-3 pathways [18,63,64]. Of importance, constitutive phosphorylation of IGF1R constitutes a universal feature of all (or most) malignantly transformed cells.

**Figure 2:**
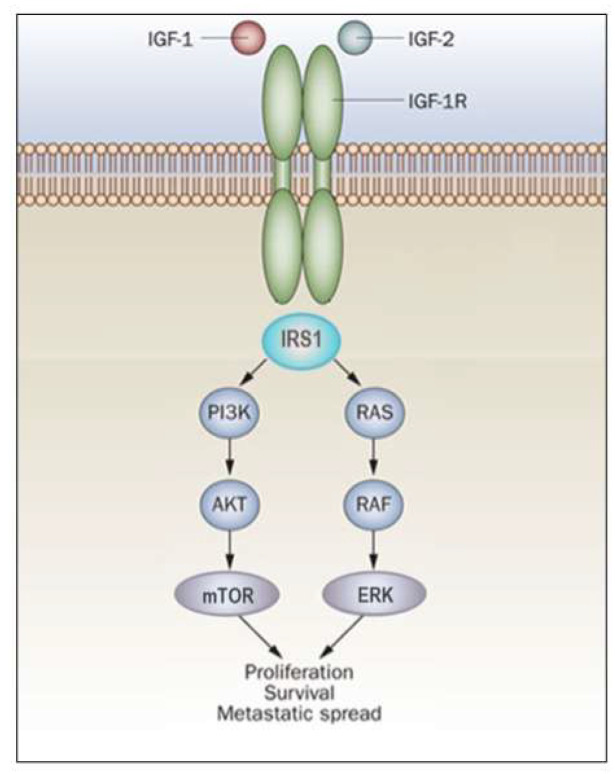
**IGF1R signal transduction**. The biological actions of IGF1 and IGF2 are transduced by the IGF1R and moderated by a family of at least six IGFBPs. Ligands bind with high affinity to the extracellular portion of IGF1R, and stimulate autophosphorylation of its tyrosine kinase (TK) domain. Upon activation of the IGF1R, IRSs become phosphorylated, with ensuing activation of two cascades, the RAS-MAP kinase (or ERK) and the PI3K-PDK1-Akt/PKB networks. The net consequence of the activation of these pathways is a boost in proliferation and a decrease in apoptosis.

In general, activation of the MAPK pathway leads to an increase in proliferation. On the other hand, activation of PI3K inhibits apoptosis and stimulates protein synthesis [65]. Phosphorylated IRS1 activates the 85-kDa regulatory subunit of PI3K, with ensuing activation of various downstream substrates, including AKT/PKB. In turn, AKT phosphorylation stimulates protein synthesis via mTOR activation, and elicits the anti-apoptotic effects of IGF1R via inactivation of BAD. In parallel, recruitment of Grb2/SOS by phosphorylated IRS1 or Shc leads to recruitment of Ras, with ensuing activation of the Ras-MAPK pathway. In addition to these networks, IGF1R signals also through the Janus kinase/signal transducer and activator of transcription pathway (JAK/STAT). Phosphorylation of JAK proteins leads to activation of STAT proteins. In particular, activation of STAT3 is critical for the potentially transforming activity of IGF1R. JNK kinases are also activated by IGF1R. IGF1 exerts inhibitory activities on JNK activation via phosphorylation and inhibition of MAP3K5/ASK1, which directly associates with IGF1R [66,67]. A simplified version of the signal transduction events mediated by IGF1R is shown in Figure 2.

Of major biological relevance, the substantial majority of the components of the signaling networks described above are shared by both IGF1R and InsR. This finding raises the question of how these receptors succeed in engaging in radically different biological activities despite a major overlap in their signaling molecules [14,68]. Several mechanisms were postulated to explain this paradox, including different distributions of InsR and IGF1R in tissues and organs, different subcellular distribution of the hormone-receptor complex, different internalization kinetics [69] and different hormone–receptor affinities [70,71,72]. In addition, various substrates and signaling mediators that are preferentially activated by either insulin or IGF1 have been identified. For example, the adapter protein Grb10 associates mainly with InsR, but not with IGF1R [73]. Likewise, the InsR, but not the IGF1R, interacts with pp120 [74]. Hence, differential activation of these and other substrates may explain, at least in part, the specificities of IGF1R and InsR.

## 6. Nuclear Import of IGF1R

Besides its typical mechanism of action at the cell-surface level, IGF1R is capable of translocating to the cell nucleus after modification by small ubiquitin-like modifier protein (SUMO)-1 [75,76,77,78]. Nuclear translocation is usually regarded as a ligand-dependent process, although some reports have provided evidence that mobilization of the cell-surface receptor may also take place in the absence of IGF1 stimulation. Nuclear IGF1R displays a number of activities that are classically correlated with transcription factors. These actions include DNA binding in a sequence-specific manner and transcription control [79,80]. Electrophoretic mobility shift assays in combination with super-shift assays using an IGF1R antibody allowed Sehat et al. to establish that IGF1R physically interacts with DNA [76]. The capacity of IGF1R to interact with DNA was investigated at a genome-wide level using chromatin immunoprecipitation assays. The majority (~80%) of IGF1R-enriched regions were intergenic (i.e., distal from annotated genes), whereas ~6% of these regions were present in introns and ~6% in exons. Data are in agreement with the idea that IGF1R binds to enhancer elements and functions as a transcription factor.

The finding that IGF1R migrates to the cell nucleus and interacts with DNA in a sequence-specific fashion supports the notion that in addition to the prototypic activities elicited by the receptor, IGF1R modulates biological processes at a genomic level [81]. Furthermore, while nuclear IGF1R’s presence was initially described in tumor cells (and, accordingly, inferred to constitute a pathologic type of localization), recent analyses have demonstrated that a pattern of nuclear IGF1R presence has also been seen in non-malignant human cells, including primary fibroblasts [82,83].

The potential consequences of nuclear IGF1R import in the clinic is a topic of great relevance [84]. While studies have shown that elevated nuclear IGF1R staining correlated with an adverse prognosis, the mechanisms responsible for nuclear IGF1R-mediated proliferation have not yet been investigated [85]. Furthermore, whilst inhibition of nuclear IGF1R migration by clathrin inhibitors correlated with major reductions in cell proliferation and invasiveness, the proteins that interact with IGF1R in the nucleus are yet to be identified [77,81]. Of major relevance, the discovery that MAPK, a key IGF1R cytoplasmic target, undergoes in itself nuclear translocation, raises questions regarding the mechanism/s of action of the IGF1R/MAPK intracellular signaling network [86,87,88]. In particular, it will be important to assess whether the nuclear transport of the cell-surface receptor and its cytoplasmic mediator take place in a coordinated fashion. Additional emerging questions include the following: (1) what is the functional significance of the joint nuclear localization of both IGF1R and MAPK?; and (2) does the pattern of IGF1R-MAPK nuclear migration reflect a generalized paradigm in cellular signaling?

## 7. Interaction of IGF1R with the p53 Genome Protection Axis

IGF1R expression is an important prerequisite for malignant transformation. Consistent with this concept, fibroblasts (termed R-) derived from *igf1r* knock-out mice are resistant to transformation by any of a series of oncogenes, including the SV40 large T antigen, activated *ras*, etc. [89,90]. However, introduction of a construct expressing a functional IGF1R into R- cells renders them sensitive to transformation. Nevertheless, IGF1R expression seems not to be an obligatory prerequisite, as suggested by the fact that various oncogenes induce transformation through alternative, IGF1R-independent pathways. In general, malignant cells exhibit augmented numbers of IGF1R on their cell surface as well as high levels of *IGF1R* mRNA. Constitutive activation (phosphorylation) of the receptor is regarded as a universal feature of cancer cells.

In addition to its mitogenic potential, the strong anti-apoptotic competence of IGF1R is undoubtedly the single most important trait that allows the receptor to play a key role in transformation. IGF1R protects cells from apoptotic death in multiple types of cultured cells as well as in vivo [91,92]. Increased concentrations of IGFIR at the cell-surface allowed cells to switch from a ‘*non-mitogenic*’ to a ‘*mitogenic*’ mode. Above a certain limit, cells acquired the ability to propagate in soft agar, a parameter of invasiveness [93,94]. It is relevant to question how terminally differentiated cells succeed in reducing IGF1R expression and, as a result, remain in a quiescent state. A potential mechanism that might be directly responsible for keeping IGF1R levels below a certain limit involves its transcriptional suppression by anti-oncogenes or tumor suppressors.

Tumor suppressor p53, the most frequently mutated molecule in human cancer, suppresses *IGF1R* promoter activity by ~90% as well as *IGFIR* mRNA levels [95]. In contradistinction, tumor-derived forms of p53 that result from mutations at codons 143, 248 or 273 enhanced promoter activity by ~2–4-fold [96]. The mechanism of action of p53 involves protein–protein interactions between p53 and components of the basal transcription machinery, including transcription factor Sp1. Extensive data indicate that the effects of p53 on cell cycle arrest are partly mediated by transcriptional suppression of the strongly anti-apoptotic *IGF1R* gene. Lack of inhibition of the *IGF1R* promoter by mutant p53 forms leads to a reduction in apoptosis, thus conferring an augmented survival capacity to cancer cells [97,98].

The identification of the *IGF1R* gene as a downstream target of p53 provides a plausible paradigm with potentially important relevance in cancer biology. Is this model shared by other members of the p53 family? We have previously evaluated the hypothesis that p63/p73 proteins, similarly to p53, control cell proliferation via mechanism/s that involve modulation of *IGF1R* expression [99]. Data show that both proteins downregulate *IGF1R* promoter activity as well as endogenous IGF1R protein abundance. On the other hand, mutated molecules were weakened in their ability to suppress *IGF1R* gene expression. Hence, data indicate that the *IGF1R* gene is a target for members of the p53/p63/p73 family. Reduced IGF1R levels in terminally differentiated cells correlate with a decrease in the extent of IGF-mediated biological effects. Deregulated expression of the *IGF1R* gene in cancer cells by mutated p53/p63/p73-mediated pathways might be linked to defective checkpoint arrest and augmented transforming capacity.

## 8. Therapeutic Opportunities

**Figure 3:**
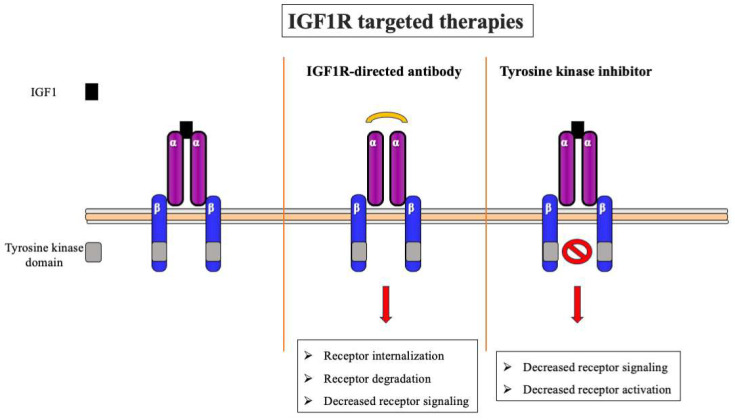
**IGF1R-targeted therapies**. Schematic representation of two of the most common approaches for IGF1R targeting: anti-IGF1R monoclonal antibodies (**center**) and small-molecular-weight IGF1R tyrosine kinase inhibitors (**right**). Blocking of IGF1R by specific antibodies (usually against the extracellular domain) leads to a decrease in ligand binding and IGF1R activation with ensuing receptor internalization and degradation. Tyrosine kinase inhibitors abrogate IGF1R activation and signaling, without major effects on IGF1R expression.

The IGF axis is an attractive therapeutic target in oncology [18,100,101,102]. Three major classes of compounds that have been studied in vitro, in animal models, and in clinical trials are (1) antibodies directed against IGF1R; (2) small-molecular-weight tyrosine kinase inhibitors; and (3) antibodies directed against IGF ligands [103] (Figure 3). Additional therapeutic modalities include the use of antisense oligonucleotides, small interfering RNA, etc. [104]. The targeting of IGF1R with a specific antibody has been the most pursued method for abrogating IGF signaling in clinical investigations to date. IGF1R antibodies block signaling via two different mechanisms: (1) the prevention of ligand binding; and (2) the induction of receptor internalization and degradation [105]. In vitro and preclinical models provided evidence that monoclonal antibodies targeting the IGF1R inhibit IGF1/2-stimulated proliferation of different solid tumors and certain hematologic cancers [106,107]. Furthermore, the anti-tumoral effect of IGF1R antibodies has been augmented by combined treatment with cytotoxic drugs [108].

In recent years, a number of specific IGF1R antibodies have been developed. Some of these antibodies have been evaluated in Phase I and II clinical trials as monotherapy, as well as in combination with chemotherapy, radiotherapy and/or other antibodies [109,110,111]. The objective response to IGF1R-directed targeting as monotherapy was, for the most part, low [112]. Hence, IGF1R-targeted therapies are expected to augment the efficacy of cytotoxic and other biological therapies. Unfortunately, few of these IGF1R antibody trials have progressed to or completed Phase III studies. Endocrine levels of IGFs or IGFBPs might be predictive for IGF1R targeting, though it seems more likely that IGF tumor expression will have more significant predictive values of response.

Finally, it is important in this context to emphasize the involvement of the GH-IGF1 signaling axis in aging and lifespan. Biosynthesis of both hormones decreases as we age, due to the diminished activity of the hypothalamic neuroendocrine axis. Disruption of this axis correlates with extended lifespan in various species, including flies, nematodes and mice. It is believed that reduced GH-IGF1 signaling might affect nutrient sensing and response to oxidative (and other forms of) stress, with an ensuing prolonged lifespan (in animal models) and healthspan. The impact of reduced IGF1 levels in congenital IGF1 deficiencies in humans is still a matter of debate.

## 9. Conclusions

The GH-IGF1 endocrine axis has a central role in the regulation of normal growth, development, and metabolism. The Somatomedin Hypothesis, formulated in the late 1950s, postulated that the physiological effects of GH are mediated via enhanced hepatic production of IGF1. The components of the GH-IGF1 axis as well as their mechanisms of action have been extremely well characterized over the years. In addition, the clinical impact of the malfunctioning of this network has been also well documented.

The IGF1R has been identified as a highly potent anti-apoptotic, pro-survival and potentially transforming receptor. Cells expressing high levels of cell-surface IGF1Rs are expected to endure, which is a hallmark of cancer cells. These attributes positionIGF1R at a critical point in oncogenic networks. Controlling the expression of this gene appears to be a potential mechanism that allows the cell to “decide” whether to adopt proliferative or apoptotic pathways. The expression of the *IGF1R* gene is determined, to a significant extent, at the transcriptional level. Evidence has been shown that a number of anti-oncogenic agents, including tumor suppressor p53, regulate cell fate by governing the expression of the *IGF1R* gene. As alluded to above, IGF1R has emerged as a promising therapeutic target. However, it is critical to identify biomarkers that can predict responsiveness to IGF1R-directed therapies.

Finally, a better understanding of the mechanisms of action of IGF1R, including characterization of interactions with signal transduction molecules, will largely improve our ability to deliver IGF1R-targeted therapies.

## Data Availability

Not applicable.
